# Clinical Cases

**Published:** 1883-06

**Authors:** E. W. Berridge


### CLINICAL CASES.

E. W. BERRIDGE, M. D.

(1). *Syphilinum in leucorrhœa.*—Mrs. K. wrote to me about the following symptoms Sept. 27th, 1880. Acrid discharge, causing violent itching and inflammation of external organs, *worse at night* from warmth of bed; the parts are very tender. She feels *generally worse at night.* *Syphilinum* DM (F. C.) one dose.

Oct. 4th.—Writes to say that menses came on the day after the dose; the itching and inflammation were better (as usual) during menses, but returned afterward, though not nearly so badly as before. On third day after the dose, several ulcerated spots on arm, stomach, leg, and finger; she has them habitually about face, chiefly on left cheek; this increased development of them seems to be an effect of the medicine.

Nov. 4th.—Writes that the vaginal symptoms are quite cured.

(2.) *Lac Felinum in weak eyes.*—June 5th, 1877. Mrs. S.—Sight bad for more than a year, getting worse lately. *On looking fixedly or reading or writing, darting pain from eyes nearly to occiput, much worse in right eye;* this symptom has lasted for a year, and is getting worse. Crawling on top of *brain* at times for a year. Weight on vertex at times for a year. *Lac Felinum*$^{40m}$ (Fincke), one dose.

12th.—Sight better. Darting pains and crawling only noticed twice.

March 25th, 1878.—In two or three weeks after last visit she improved very much in every respect; the sight was better, and the darting very seldom occurred. Now the crawling has almost ceased for four or five months; weight on vertex has remained the same; for the last month, after overwork in nursing a patient, the sight has become worse and the darting returned. *Lac Felinum*$^{40m}$ (Fincke), one dose

April 1st.—Darting in eyes gone for five days.

8th.—Darting only once; sight better; crawling gone; weight better.

15th.—The darting was bad yesterday morning, but at no other time. Sight stronger. Crawling returned. Weight increased.

May 20th.—The darting has occurred only once, about three weeks ago, and in left eye only; sight still weak; crawling and weight nearly gone.

June 17th.—No more darting or crawling; weight bad.

Aug. 12th.—No more darting or crawling; weight much better; sight still bad.

Nov. 20th.—No return of darting or crawling; weight gone for some time; sight still bad. Has cough with thick and frothy expectoration, worse in open air. *Phosphorus* CM (F. C.), one dose.

Feb. 6th, 1879.—Sight better, but weak. No return of darting, crawling, or weight. No further report.

This patient was an elderly monthly nurse, and her avocations were a great hindrance to her recovery.

(3.) *Lac Felinum in weak eyes.*—Dec. 5th, 1878.—Miss M., æt. 26. Sight impaired for four or five years; could not see to read, with pain at back of eyes. Now, *when reading*, the letters run together, and there is dull, aching pain behind eyes, or *shooting in eyes*, the confused sight and the shooting being *worse in the right eye.* At times, for some years, mental illusion that the corners of the furniture, or any pointed object near her, were about to run into her eyes. (This symptom is purely mental; they do not appear to her sight to be too close. *Lac Felinum*$^{40m}$ (Fincke), one dose.

Dec. 17th.—Has tried eyes as much as usual. Less confusion of sight, very little aching, no shooting. Illusion better, only noticed when sewing or knitting.

Jan. 24th, 1879.—Writes that the illusions are worse. *Lac Felinum*$^{40m}$ (Fincke), one dose.

Feb. 4th.—Pains in eyes quite gone; the shooting has never returned, and the aching has gone for a week; less confusion of sight when reading. The illusion has quite gone for a week; has been much more free from it since taking the *Lac Felinum.*

17th.—For the last few days the pains have occasionally returned, but without the illusion.

March 6th.—Only once since has had a recurrence of the shooting, with the illusion of objects running into them.

24th.—On the 20th, after over-fatigue in walking, the old symptoms returned, but this time the pains were entirely confined to the right eye; have not returned since.

April 10th.—No return of the pains, except pains in eyes when reading by gaslight, and then it is much slighter than before. Feels very much better generally.

May 6th.—Writes to say that for nearly a week she has had severe headache, with illusion as if things were running into eyes. *Lac Felinum*$^{40m}$ (Fincke), one dose.

17th.—No return of pain in head or eyes.

June 20th.—On 12th the pains returned for a day, but only in the left eye

July 12th.—No return of old symptoms, except the illusion of the needle running into eyes. *Lac Felinum*$^{40m}$ (Fincke), a dose every day for seven days.

Aug. 16th.—Writes that since taking the last powders the old symptoms have returned; neuralgia in face and general weakness for more than a week.

As the repetition of the 40$^{m}$ potency seemed to have caused an aggravation, I sent *Lac Felinum*$^{cm}$ (Fincke), a powder every day for seven days.

Aug. 30th.—Since taking the powders, no return of neuralgia; feels much better generally; eyes still trouble her at times, but no pain in head.

Oct. 3d.—Has had two slight attacks of the pains in the head and eyes.

Nov. 17th.—About the beginning of this month had a violent bilious attack, for which she took *Nux* on her own account. During this attack the old symptoms returned with great violence, and have frequently occurred since. *Lac Felinum*$^{cm}$ (Fincke), a daily dose for seven days.

27th.—No return of pains for the last five days.

Jan. 6th, 1880.—Has caught a severe cold, with return of symptoms. *Lac Felinum*$^{cm}$ (Fincke), a daily dose for seven days.

April 5th.—Writes that the eye symptoms, though greatly relieved, return every time she takes cold or is over-fatigued. No further report.

This case, though not thoroughly cured, shows the marked action of *Lac Felinum* on the eyes. I have also cured with this remedy a case of ulceration of the cornea pronounced incurable by an allopath.

(4.) *Hydrophobinum in enuresis.*—Miss S. yesterday and to-day complained of constant desire to urinate *on seeing running water,* urinating only a little at a time. At 12.40 P. M. I gave her one dose of *Hydrophobinum*$^{cm}$ (Swan). Within an hour she was much better and was soon well.

This case, which verifies similar cases reported by other observers

(see *Hahnemann's Monthly*, vol. I, p. 220, and *American Hom. Review*, vol. IV, p. 486) is important, because the characteristic aggravation, though an almost invariable condition of hydrophobia, has not yet been produced by the dynamized nosode. This proves that the symptoms of a disease which can be *clearly* traced to the morbific action of a specific virus should be incorporated in the pathogenesis of the corresponding nosode. Thus the symptoms of all cases of hydrophobia and anthrax ought to be united to the clinical and pathogenetic effects of the dynamized *Hydrophobinum* and *Anthracinum*, as C. Hering has done.

The great difficulty, however, is to ascertain beyond the shadow of a doubt which symptoms are due to the virus itself and which to other morbid taints previously in the patient's system and roused to manifestation by it. The more acute and self-limited the disease, the less will be the admixture of its own peculiar symptoms with those of others, and *vice versa*. Thus to incorporate all the life-long effects of syphilis in a pathogenesis of *Syphilinum* would be an error, because many of them might be really due to psora or improper drug-treatment; and this idea is confirmed by the fact that *Syphilinum* will *not* cure every case of syphilis, probably owing to these complications.

Nevertheless, even these must not be absolutely rejected. One of Dr. Swan's patients, a man suffering from tertiary syphilis, complicated with drug-treatment (mercurialization, etc.), had the following symptoms: "Pains commence at, and increase gradually after, 2 P. M., reach their height at 9 P. M., and continue exceedingly acute till the following 3 or 4 A. M., *and with the break of day they subside.*" This symptom, though necessarily doubtful at first, has been proved by repeated clinical experience to be *the grand characteristic* of this remedy, and I have recently confirmed its true syphilitic origin in Dr. Swan's patient by a fragmentary proving with a high potency of *Syphilinum*. Provings and clinical experience with potencies will clear up all these difficult problems, and they should be collected and augmented as soon as possible. In my opinion, the virus of each contagious disease (hydrophobia, syphilis, etc., etc.) is one and indivisible, and the variously differing effects which we see them produce are due, not to a difference in different specimens of the virus itself, but to the ever-varying constitutions of the recipients of them. Thus the strictest individualization must be resorted to in every case, if we would wish to be uniformly successful in their treatment.

(8.) *Bromine in headache.*—Aug., 1881.—Mrs. B. B. complained of throbbing pain in left temple, worse on stooping. The only remedy given in my MS. Head Repertory as possessing this symptom is *Bromine;* and a single dose of the CM (Fincke) potency cured.

(9.) *Silicea in vertigo.*—Aug. 12th, 1881.—Miss L. since 4.30 A. M. has had vertigo, as if all things were going round; worse on lying, especially when lying on the left side; better by standing up, but returning soon after lying down again. At 8.40 A. M. I gave her one dose of *Silicea*$^{cm}$ (F. C.). After this the vertigo did not return, even when lying. *Silicea* is the only remedy known to produce vertigo *from lying on the left side* (see symptom 101 in Allen). It was produced on the prover by the 4th, 3d, and 2d dilutions, and cured by the CM dilution.

---

